# Supplementary material for: Pollen trapping as Honeybees pollination management and identification of dominant pollinators of Guizotia abyssinica (L.f)
Source: PLoS One. 2024 Oct 10;19(10):e0292565. doi: 10.1371/journal.pone.0292565 (PMC11469591; doi:10.1371/journal.pone.0292565)
Supplement: Attachment — Submitted filename: PONE-D-23-28714.pdf [file pone.0292565.s001.pdf]

PLOS ONE

Pollen Trapping As Honeybees Pollination Management and Identification of a Dominant Pollinators of Guizotia abyssinica L.

-Manuscript Draft--

Manuscript Number:	PONE-D-23-28714
Article Type:	Research Article
Full Title:	Pollen Trapping As Honeybees Pollination Management and Identification of a Dominant Pollinators of Guizotia abyssinica L.
Short Title:	Pollen Trapping As Honeybees Pollination Management
Corresponding Author:	Kasim Roba Jilo, Msc<br>Ethiopian Institute of Agricultural Research<br>Holeta, Oromiya ETHIOPIA
Keywords:	Dominant Pollinators , Guizotia abyssinica L, Honeybees, Identification, Pollen trapping, Pollination management
Abstract:	Pollination is one of the most fascinating aspects of insect-plant interactions. Pollen is the male reproductive element of flowering plants gathered by foraging field bees from the male parts of flowering plants called the anther. Guizotia abyssinica (L.f) is an important oilseed crop cultivated in Ethiopia and India which belongs to family Asteraceae. Though self-incompatibility is found in Guizotia abyssinica, a higher seed set is experienced in place with an active Honeybees population. To improve seed yield of Guizotia abyssinica agricultural practices usually focus on inputs,such as, fertilizer application; however, have litle effect on yield if availability of insect pollination is too low. To fill this gap an experiment was carriedout at Dandi district, West Shoa zone, Oromia,Ethiopia to see the Honeybees pollination management as an agronomic inputs. Pollination management of Honeybees was tested under different colony managements: Pollen trapping with and without sugar syrup feeding. Results discovered that from Honeybees fed sugar syrup much pollen was collected more than colonies not fed sugar syrup. The proportion of Guizotia abyssinica pollen collected through sugar syrup feeding of colony was greater (62.2 %), compared to the proportion pollen trapped without sugar syrup feeding (37.8%). This indicates that sugar syrup feeding enhances the collection of pollen and probability this enhances pollination efficiency of Honeybees since they visit frequently to fulfill their daily protein requirement from pollen. Therefore, Honeybees pollination services should be included as one of agronomic input to meet the yield of Guizotia abyssinica with sugar syrup feeding as pollination management.
Order of Authors:	Kasim Roba Jilo, Msc

Powered by Editorial Manager® and ProduXion Manager® from Aries Systems Corporation

	Gemmechis legesse Yedata, MSc
	Tolera Kumsa Gemmeda, PhD
Additional Information:	
Question	Response
Financial Disclosure<br><br>Enter a financial disclosure statement that describes the sources of funding for the work included in this submission. Review the submission guidelines for detailed requirements. View published research articles from PLOS ONE for specific examples.<br><br>This statement is required for submission and will appear in the published article if the submission is accepted. Please make sure it is accurate.<br><br>Unfunded studies<br>Enter: The author(s) received no specific funding for this work.<br><br>Funded studies<br>Enter a statement with the following details:<br>• Initials of the authors who received each award<br>• Grant numbers awarded to each author<br>• The full name of each funder<br>• URL of each funder website<br>• Did the sponsors or funders play any role in the study design, data collection and analysis, decision to publish, or preparation of the manuscript?<br>• NO - Include this sentence at the end of your statement: The funders had no role in study design, data collection and analysis, decision to publish, or preparation of the manuscript.<br>• YES - Specify the role(s) played.	funded for data collection only

* typeset	
Competing Interests<br><br>Use the instructions below to enter a competing interest statement for this submission. On behalf of all authors, disclose any competing interests that could be perceived to bias this work—acknowledging all financial support and any other relevant financial or nonfinancial competing interests.<br><br>This statement is required for submission and will appear in the published article if the submission is accepted. Please make sure it is accurate and that any funding sources listed in your Funding Information later in the submission form are also declared in your Financial Disclosure statement.<br><br>View published research articles from PLOS ONE for specific examples.	26761
NO authors have competing interests<br><br>Enter: The authors have declared that no competing interests exist.<br><br>Authors with competing interests<br><br>Enter competing interest details beginning with this statement:<br><br>I have read the journal's policy and the authors of this manuscript have the following	

Powered by Editorial Manager® and ProduXion Manager® from Aries Systems Corporation

competing interests: [insert competing interests here]<br><br>* typeset	
Ethics Statement<br><br>Enter an ethics statement for this submission. This statement is required if the study involved:<br><br>• Human participants<br>• Human specimens or tissue<br>• Vertebrate animals or cephalopods<br>• Vertebrate embryos or tissues<br>• Field research<br><br>Write "N/A" if the submission does not require an ethics statement.<br><br>General guidance is provided below. Consult the submission guidelines for detailed instructions. Make sure that all information entered here is included in the Methods section of the manuscript.	not animal included

Format for specific study types<br><br>Human Subject Research (involving human participants and/or tissue)<br>• Give the name of the institutional review board or ethics committee that approved the study<br>• Include the approval number and/or a statement indicating approval of this research<br>• Indicate the form of consent obtained (written/oral) or the reason that consent was not obtained (e.g. the data were analyzed anonymously)<br><br>Animal Research (involving vertebrate animals, embryos or tissues)<br>• Provide the name of the Institutional Animal Care and Use Committee (IACUC) or other relevant ethics board that reviewed the study protocol, and indicate whether they approved this research or granted a formal waiver of ethical approval<br>• Include an approval number if one was obtained<br>• If the study involved non-human primates, add additional details about animal welfare and steps taken to ameliorate suffering<br>• If anesthesia, euthanasia, or any kind of animal sacrifice is part of the study, include briefly which substances and/or methods were applied<br><br>Field Research<br><br>Include the following details if this study involves the collection of plant, animal, or other materials from a natural setting:	

Powered by Editorial Manager® and ProduXion Manager® from Aries Systems Corporation

• Field permit number<br>• Name of the institution or relevant body that granted permission	

Powered by Editorial Manager® and ProduXion Manager® from Aries Systems Corporation

Data Availability<br><br>Authors are required to make all data underlying the findings described fully available, without restriction, and from the time of publication. PLOS allows rare exceptions to address legal and ethical concerns. See the PLOS Data Policy and FAQ for detailed information.	Yes - all data are fully available without restriction
A Data Availability Statement describing where the data can be found is required at submission. Your answers to this question constitute the Data Availability Statement and will be published in the article, if accepted.<br>Important: Stating 'data available on request from the author' is not sufficient. If your data are only available upon request, select 'No' for the first question and explain your exceptional situation in the text box.<br><br>Do the authors confirm that all data underlying the findings described in their manuscript are fully available without restriction?	

Powered by Editorial Manager® and ProduXion Manager® from Aries Systems Corporation

Describe where the data may be found in full sentences. If you are copying our sample text, replace any instances of XXX with the appropriate details.<br><br>• If the data are held or will be held in a public repository, include URLs, accession numbers or DOIs. If this information will only be available after acceptance, indicate this by ticking the box below. For example: All XXX files are available from the XXX database (accession number(s) XXX, XXX.).<br>• If the data are all contained within the manuscript and/or Supporting Information files, enter the following: All relevant data are within the manuscript and its Supporting Information files.<br>• If neither of these applies but you are able to provide details of access elsewhere, with or without limitations, please do so. For example:<br><br>Data cannot be shared publicly because of [XXX] . Data are available from the XXX Institutional Data Access / Ethics Committee (contact via XXX) for researchers who meet the criteria for access to confidential data.<br><br>The data underlying the results presented in the study are available from (include the name of the third party	26761

and contact information or URL).<br>• This text is appropriate if the data are owned by a third party and authors do not have permission to share the data.<br><br>* typeset	
Additional data availability information:	Tick here if the URLs/accession numbers/DOIs will be available only after acceptance of the manuscript for publication so that we can ensure their inclusion before publication.; Tick here if your circumstances are not covered by the questions above and you need the journal's help to make your data available.

Powered by Editorial Manager® and ProduXion Manager® from Aries Systems Corporation

original work Click here to access/download;Manuscript;JOURNAL OF GUIZOTIA.docx

Pollen Trapping As Honeybees Pollination Management and Identification of a Dominant Pollinators of *Guizotia abyssinica* L.

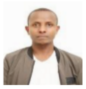

Kasim Roba, Gemmechis Legessa, Tolera Kumsa

Holeta Bee Research Center, P.O. Box-22, Holeta, Ethiopia

Corresponding author email: kasimroba7@gmail.com, phone number +251927983536 or +251935356813

Abstract

Pollination is one of the most fascinating aspects of insect-plant interactions**.** Pollen is the male reproductive element of flowering plants gathered by foraging field bees from the male parts of flowering plants called the anther. *Guizotia abyssinica* (L.f) is an important oilseed crop cultivated in Ethiopia and India which belongs to family Asteraceae. Though self-incompatibility is found in *G~~uizotia~~ abyssinica*, a higher seed set is experienced in place with an active Honeybees population. To improve seed yield of *G~~uizotia~~ abyssinica* agricultural practices usually focus on ~~inputs,such as,~~ fertilizer application; however, have ~~litle~~little effect on yield if availability of insect pollination is too low. To fill this gap an experiment was ~~carriedout~~carried out at Dandi district, West Shoa zone, ~~Oromia,Ethiopia~~Oromia, Ethiopia to see the Honeybees pollination management as an agronomic inputs. Pollination management of Honeybees ~~was tested~~was tested under different colony managements: Pollen trapping with and without sugar syrup feeding. Results discovered that from Honeybees fed sugar syrup much pollen was ~~collected more~~collected more than colonies not fed sugar syrup. The proportion of ~~*Guizotia*~~ *G. abyssinica* pollen collected through sugar syrup feeding of colony was greater (62.2 %), compared to ~~the proportion~~ pollen trapped without sugar syrup feeding (37.8%). This indicates that sugar syrup feeding enhances the collection of pollen and ~~probability~~ probabily this enhances pollination efficiency of Honeybees since they visit frequently to fulfill their daily protein requirement from pollen. Therefore, Honeybees pollination services should be included as one of agronomic input to meet the yield of *Guizotia abyssinica* with sugar syrup feeding as pollination management.

Keywords: Dominant Pollinators , *Guizotia abyssinica* L , Honeybees, Identification, Pollen trapping, Pollination management

Commented [Ofijan1]: Can we say pollination management?

Commented [Ofijan2]: Without yield data how you could conclude? Please incorporate its yield if you have unless exclude about the yield increment through pollination.

Introduction

Pollinators are a functional group with high relevance for ensuring cross-pollination in wild plant populations and yields in major crops (Steffan-dewenter and Westphal 2008). Pollination is one of the most fascinating aspects of insect-plant ~~interactions(~~interactions (Veereshkumar K. M. Kumaranag 2021).

Insects are one of the ecosystem services that are very beneficial for human life and other living things (Nurlaila *et al.* 2021). Insects, especially bees, are important in bringing about the pollination of many plant species and in temperate regions attention has been given to the deliberate manipulation of these pollen vectors so as to increase crop yields (Sc 2016). *Apis mellifera* is principal insect species used for pollination of crops (Eeraerts *et al.* 2019). Bees have a trichromatic visual system that is sensitive to green, ultraviolet, white, and blue wavelengths, allowing them to see numerous colors (Shaden *et al.*, 2021). Apis mellifera is also relatlvely polytroplc and for these reasons it is especially suitable for the purposes of Pollen vector manipulation (Sc 2016).

Pollination plays a vital role in maintaining the natural balance of ecosystems and is the cornerstone of crop production, providing a link between agriculture and the cycle of life (Shaden *et al.* 2021). Nectar composition (% sucrose) and total nectar sugar concentration (% sugar) has relation to pollination syndrome (T. Kromer1, M.Kessler1 2008). Pollination is the movement of pollen from anthers of a flowers to the stigma of the same or a different flower (Foundation for Arable Research 2012).

It is common practice among ecologists to complete the description of a community by one or two numbers expressing the "diversity" or the "evenness" of the community (Carlo H.R. HElP *et al.*,1998). From the applied perspective, species richness is the ultimate ‘‘score card’’ in efforts to preserve biodiversity in the face of increasing environmental pressures and climate change resulting from human activity (Gotelli and Chao 2018). Species richness, evenness and biodiversity and their common methods of measurement (evenness index: Simpson, Camargo and Smith & Wilson; biodiversity index: Shannon-Wiener, Brillouin and Simpson and it is important to know the suitable index and its measurement method to study species diversity (Goudarzian and Sy 2017).

G.abyssinica (L.f) Cass. is an important oilseed crop cultivated in Ethiopia and India widely which belongs to the family Asteraceae, tribe helianthus, and sub-tribe coreopsinidae grown over an area of 232.1 thousand hectares with an average production of 76.2 thousand tones in India (Kachhela and Pastagia 2018). The genus Guizotia belongs to the family of Compositae, tribe Heliantheae, subtribe Coreopsidinae (Sharma 2014).

Global declines in pollinator diversity and abundance have been recognized, raising concerns about a pollination crisis of crops and wild plants (Ormann and Olzschuh 2013). Honey bees (Apis mellifera) and non-Apis bees in the United States has grown increasingly important, because declines in their populations have the potential to impact food security due to loss of pollination services (Statement 2017). For this reasons to over come the problems of yield lose obtained form pollination services ,pollination management is important and that is why this research was done on pollination management of honeybees on *G.abyssinica L.*

Commented [Ofijan3]: Write either Honeybees or Honey bees consistently

Commented [Ofijan4]: A.mellifera through all and italic

Species Richness Estimation

Commented [Ofijan5]: Omit the subtitle under introduction and write its information as a paragraph

A simple count of the number of species in a sample is usually a biased underestimate of the true number of species, simply because increasing the sampling effort inevitably will increase the number of species observed (Gotelli and Chao 2018). In nature, only five percent of the crops are selfpollinated and remaining 95% are cross-pollinated, in which 10% depend upon wind and 85% on animal pollination (Tewari GN, Singh K ,1983), in which insect pollination alone accounts 90% of animal pollination (Buchmann LS, Nabhan PG (1996). Species richness is a diversity of order 0 (which means it is completely insensitive to species abundances)(Gotelli and Chao 2018). Many species diversity indices can be converted by an algebraic transformation to Hill numbers(Gotelli and Chao 2018).

Pollination is one of the most important mechanisms in the maintenance and conservation of biodiversity and in general life on earth. Pollination also benefits society by increasing food security and improving livelihoods, hence pollinator diversity is important (Thakur 2012). Worldwide, 90 percent food supply is contributed by 82 commodities assigned to plant species and bees are pollinators of 63 (i.e.70%) of these plant species and are the most important known pollinators of 39 (48%) of these plant species (Thakur 2012). Bees are keystone organisms that sustain human life on earth through their pollination services. However, very little is known about functional groups and indicator species of bee communities from agricultural landscapes in Sub-Saharan Africa (Munyuli 2012).

Commented [Ofijan6]: Compile with the next paragraph and rewrite as a paragraph since they talk about pollination

Pollination is one of the most fascinating aspects of insect-plant interactions (Veereshkumar K. M. Kumaranag 2021)**.** Pollen is the male reproductive element of flowering plants and is gathered by foraging field bees from the male parts of flowering plants called the anthers (Somerville,2012). Effects of soil quality on the interactions of plants with beneficial arthropods, such as pollinators, have not been extensively examined (Cardoza *et al.*, 2012).

In addition to broadening the range of potential pollinators based on their activity patterns, circadian trapping endows other selective advantages, including the possibility of an extended staminate phase to promote pollen deposition, and enhanced interfloral movement of pollinators (Lau *et al.*, 2017). The Apis mellifera has occupied dominating position in commercial pollination around world because this is

highly social bees (Thakur 2012). Pollination is a vital step in the reproduction of flowering plants which leads to fertilization and fruit/ seed setting (Thakur 2012).

Though self-incompatibility is found in niger, a higher seed set is experienced in place with an active bees population. This necessitates the immediate requirement of developing high self-compatible populations to enhance seed yield (Kumar *et al.*,2006). Pollens from different floral species can vary significantly in protein (amino acids), lipids/ fats, and mineral and vitamin content. For this reason, some pollen sources are considered of higher value than others in their contribution to honey bee nutrition (Somerville ,2012). A strong productive colony may consume over 50kg of pollen per year when it is being manipulated and worked intensively (Somerville,2012).

Pollen trapping should only be considered when the colony is strong and there is more than enough pollen available to meet the colony's immediate needs,Pollen varies in moisture content from between 7 and 21% when collected (Somerville,2012). Crop pollination generally increases with pollinator diversity and wild pollinator visitation (Eeraerts *et al.*, 2019).

Materials and Methods

The study was conducted in Dandi district at Ginchi, West Shoa, Oromia regional state, Ethiopia in 2020-2022. Colonies were established~~stablished~~ for pollination of *G. abyssinica* for yield increment through sugar syrup feeding as pollination management of honeybees as agronomicinputs ~~imputs~~. The flower visitors were collected with fine mesh sweep nets and got identified following ~~follwing~~ methods used by(Vanitha and Raviprasad 2019). Species evenness (or relative species abundance) in a community is another factor that affects diversity. One of the most frequent methods of evenness measurement is Simpson. Simpson's evenness index (Goudarzian and Sy 2017).

Pinning collected Bees

Diversity of species collected and added to alcohol~~alhocol~~ for preservation and brought from the experimental area, mounted , pinned for identification and then taking the dried specimens and placing them on a foam pad such that they are either upside down or on their sides were used following (Azzu 2016).

Commented [Ofijan7]: How you could talk about yield increment without data? Please don't talk about yield increment unless you have included yield data

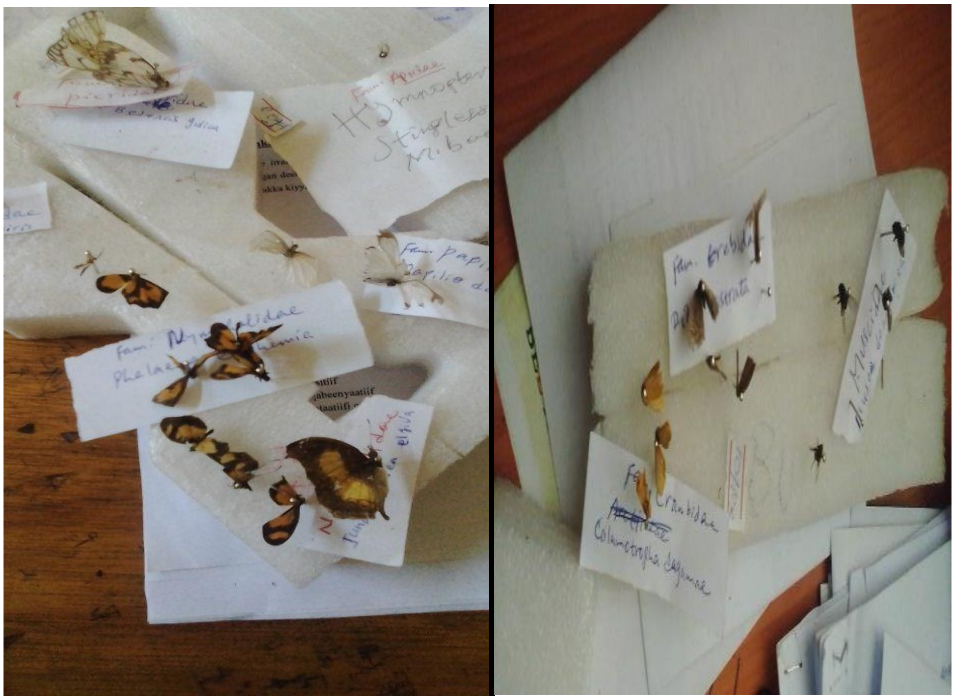

Figure 1. Mountaining and labeling of insects specimen for identification

Pollen trapping

Worker bees collected pollen from flowers and carry it back to the hive packed in pellets on the pollen baskets on the rear legs. By encouraging returning field bees to enter the hive through small holes in a punched plate, the pellets of pollen can be scraped from the legs and collected in a suitable tray (Somerville 2012). ~~196.342 gram of *G. abyssinica* was trapped by pollen trap as pollination management to enhance bees visiting frequency to increase pollination purpose and these pollens were sorted into different colors and identified to species levels and they were classified under eight species.~~ Removal of pollen or pollen trapping~~tarpping~~ may led to low honey production even though we didn't see its honey production since it was not our objective,which was reported by (Hoover and Lynae 2018). Pollen was trapped from the pellets of stored pollen during periods of pollen surplus has been done following (Somerville 2012).

Commented [Ofijan8]: Should be written under the results

Commented [Ofijan9]: It is better if you omit it b/c of it's not your objective

Bee management

Special management was given for colonies used for pollen ~~trapping under~~trapping under experiment to ensure sufficient pollen and this ~~epriemnt~~ experiment was done on active and strong colonies with equal

resources like brood and all others at the same time from the same area. This was done at around September – October active period of the area ~~when pollen~~when pollen ~~avialablity~~ availability was very high and known in the area following methods used by ~~(~~Somerville (2012).

Commented [Ofijan10]: What are others? Please list them or omit others

Bee feed

Sugar ~~syrup ratio~~syrup ratio of 1:1 was used to stimulate them to collect more pollen. Pollen was collected until niger stopped pollen production and shaded.

Flower visitors

Pollinators abundance was recorded ~~when floral~~when floral density in the field. The experiment was divided into eight plots for sampling pollinators from each plot in 10 minutes each when it was bloomed 50 % - 75 % . Sampling was done the whole day ~~continuosly~~continuously until all pollinators were absent from the flowers of *G.abyssinica*. Samplings were done through visual counting and trapping of flower visitors for 10 minutes per predetermined transect of 1m^2 area, and this was repeated five times by 10 ~~mintues~~minutes gap each between two subsequent transects following ~~(~~Veereshkumar K. M. Kumaranag (2021).

Formatted: Superscript

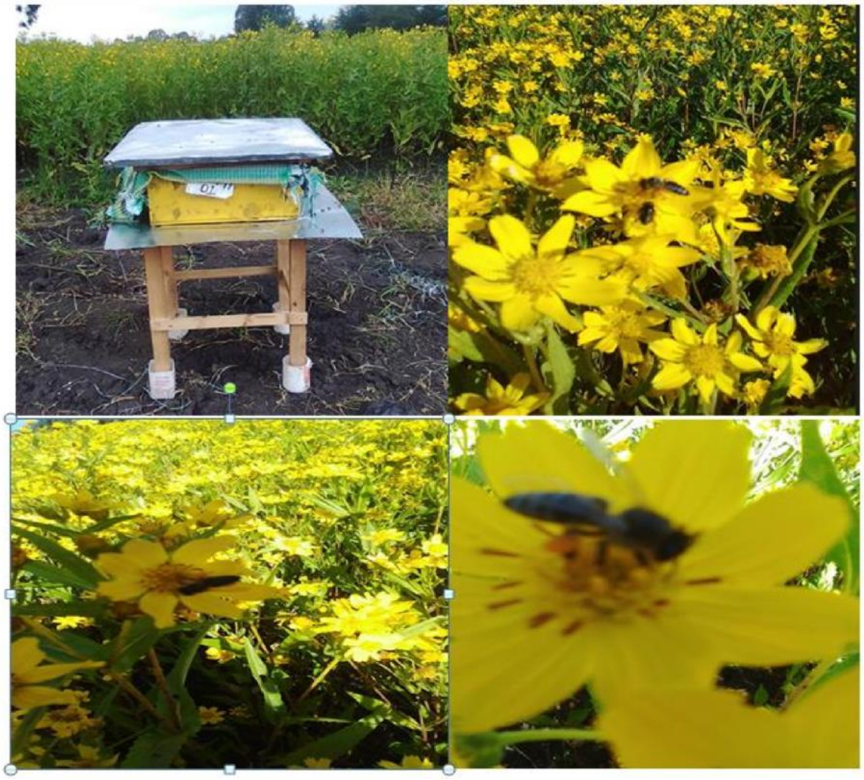

Figure ~~2 :~~**2:** Colony established for pollination purpose of ~~*Guizotia*~~ *G. abyssinica* ~~L.~~.

Honeybee colonies were established at Ginchi, Dandi district ,west Shoa ,Oromia ~~egional~~regional state, Ethiopia. Pollen trap was fitted at the entrance of bee hives having the pollen trapping efficiency of 16% and pollen loads was collected daily until it stops ~~floweing~~flowering ~~or shaded~~ . The pollen samples were placed in a clean paper bags and left for 24 hrs to dry at room temperature. The pollen grains were collected and sorted into colors and identified to the genus and species level using the pollen atlas of Ethiopia (Nuru,2002).

Data analysis

Descriptive ~~statistics ,Anova~~statistics, Anova and Shannon-Wiener Index (H') (Shannon & Weaver, 1949); were used to estimate pollinators diversity in each plots. Sugar syrup (1:1) was given ~~to each~~to each colonies by internal feeding methods ~~at Ginch~~ until the experiment was ~~completed at~~completed at every 48 hrs following methods used by ~~(~~Piano *et al.* (2021). Statistical analyses were conducted to determine the importance of some taxa relative to others. To explore whether there were statistically significant differences in occurrences or proportional abundance / species richness between different taxonomic groups (genera, families, etc), chi-square tests were applied following ~~(~~Munyuli (2012).

Maintaining the specimens

> **Commented [Ofijan11]:** Move this section at the top of data analysis

All species were identified as the same species or group grouped in rows or sections. A new row or section is started for each new taxa which, makes it easy to see the groupings. It is easiest to have a determination label precede the row or section of ~~specimens following~~specimens following methods used by ~~(~~Azzu (2016).

Pollen trapping

> **Commented [Ofijan12]:** Why you wrote the method of pollen trapping twice? Please compile into one paragraph with the previous pollen trapping method andmove at the top of data anlysis

~~Worker bees collect pollen from flowers and carry it back to the hive packed in pellets on the pollen baskets on the rear legs. By encouraging returning field bees to enter the hive through small holes in a punched tyce , the pellets of pollen can be scraped from the legs and collected in a suitable tray (Somerville 2012). Pollen collected every two days following methods used by (Somerville 2012). Traps are installed and worked continuously until the experiment ends for three weeks following methods used by (Nurlaila *et al.* 2021).~~

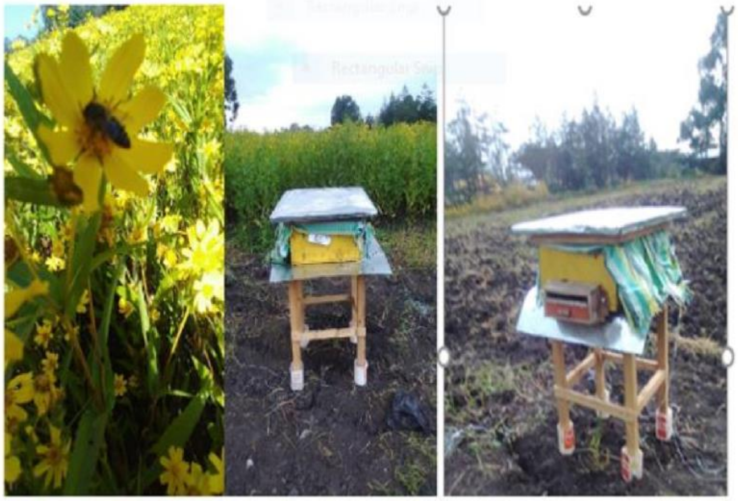

Figure 3: Hives placed at the ~~experiemal~~ experimental area attaching pollen traps on its ~~intrance~~entrance for pollen ~~tarpping~~trapping

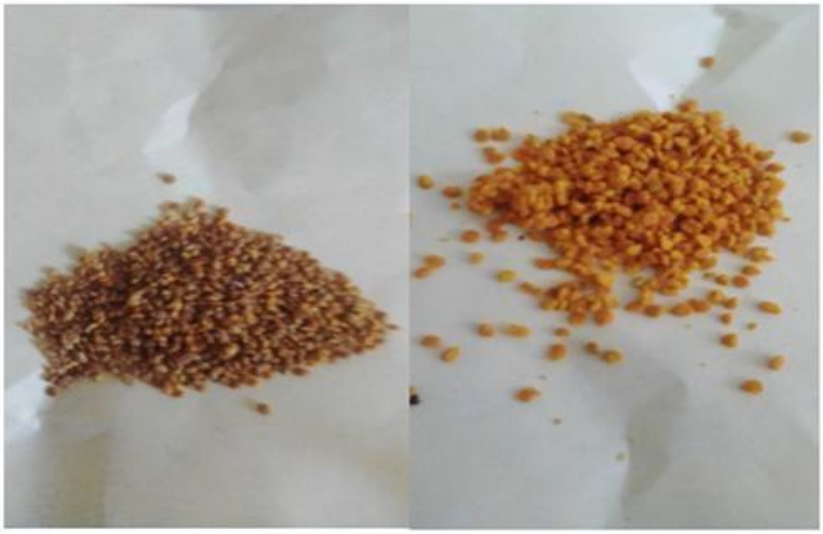

Figure 4: Unsorted and sorted pollen trapped from hives collected by Honeybees and sorted image of *G.abyssinica* ~~L.~~ pollen

Results and Discussions

Pollen weight collected by honeybees daily was much greater and significant at p-~~-~~value of 0.001 for colonies fed sugar syrup than colonies didn’t fed sugar syrup as shown in ~~(~~ Figure 5~~)~~. This implies honeybees collect more pollen when pollen trapped to fulfill their daily requirement of pollen which probably increases pollination of *G.* *abyssinica* ~~L.~~. The proportion of *G.* *abyssinica* pollen collected through sugar syrup feeding of colony for three weeks until the ~~experiemt~~experiment ended was greater (62.2 %); Compared to the proportion pollen trapped without colony feeding with sugar syrup (37.8%) .This indicates that sugar syrup feeding enhances the collection of ~~*Guzotia*~~ *G.* *abyssinica* pollen and probability the pollination efficiency of honeybees. ~~-~~Pollen weight was inferred from the pollen loads collected through pollen traps following methods used by ~~(~~Tolera *et al.*~~,~~(2018). Therefore, honey bee pollination services should be included as one of agronomic input to meet the yield of ~~*Guizotia*~~ *G.* *abyssinica* through pollination management of honeybees.

Commented [Ofijan13]: Please rewrite the sentences, is not clear

Commented [Ofijan14]: Without the yield per hectare data it’s impossible to talk about the yield increment

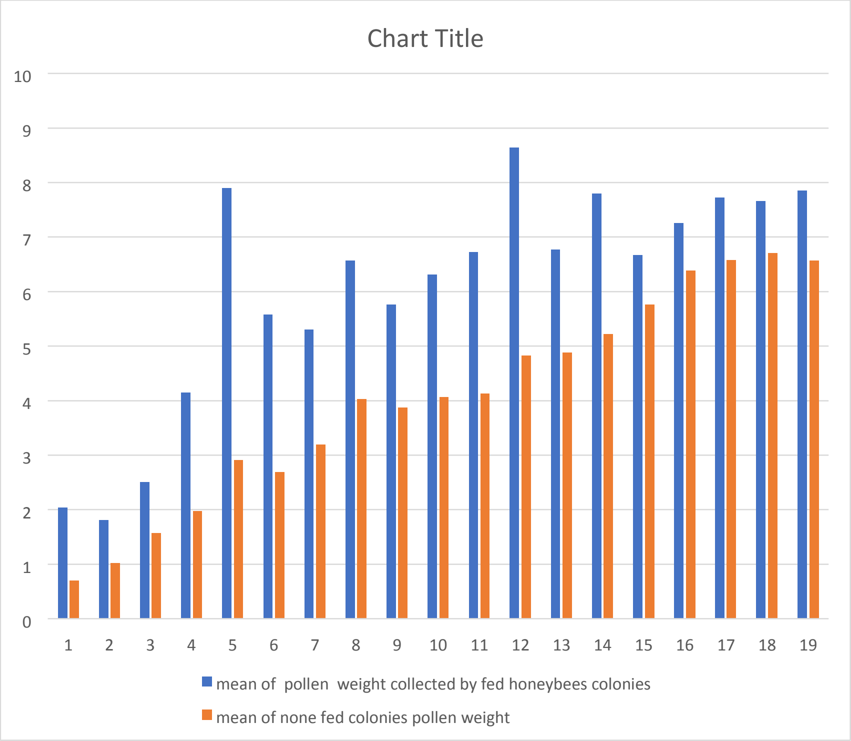

Figure 5 : Weight of pollen collected by pollen trap while pollinating *G. abyssinica*.

Commented [Ofijan15]: Please define the x and y -axis

Eight species ~~of pollen~~of pollen collected by honeybees were sorted and identified for both colonies feed sugar syrup and none feed sugar syrup as shown in Table 1 ~~below~~. ~~Feeded~~ Fed colonies collected 68.8% of ~~*Acacia abyssinica* ,~~*Acacia abyssinica*, ~~*where as*~~*whereas none* ~~*feeded*~~ *fed colonies collected* 31.3% of *A.* ~~*abyssinica which*~~*abyssinica* which has Brow Mod ~~colour~~color identified based genetics color bar code manuals. A 54.7% of *G.abyssinica* was collected by ~~feeded~~ fed colonies where as 45.3% collected by none feeded ~~colonies which~~colonies which has Neon orange Mod color. *Zantedeschia aethiopica*, *Vicia* ~~*faba* and~~*faba* and *Sesamum indicum* pollen were only collected by fed ~~colines~~ colonies which might be due to the strength of colonies collection from far areas where as *Clausena anisate* was only collected by none fed colonies in small amount which may be due to ~~weekness~~weakness of the colonies or their preference.

Formatted: Font: Italic

Formatted: Font: Italic

Formatted: Font: Italic

Formatted: Font: Italic

Formatted: Font: Italic

Formatted: Font: Italic

Table 1: Pollen trapped by ~~Honeybees ,its~~Honeybees, its color and color ~~name ,family~~name, family and species identified

Species of pollen collected	Families of species	Frequency and their percentage	Colors	Colors' names

		Not fed	fed		
Acacia abyssinica	Fabaceae	10<br><br>31.3%	22<br><br>68.8%	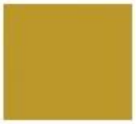	Brow Mod
Acanthus sennii	Acanthaceae	2<br><br>40%	3<br><br>60%	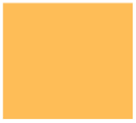	Carat Mod
Clausena anisata	Rutaceae	1<br><br>100%	0<br><br>0%	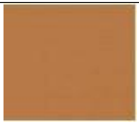	Milk Chocolate
Eucalyptus camaldulensis	Myrtaceae	13<br>30.95%	29 69.1%	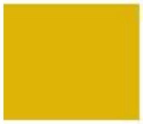	Yellow olive Mod
Guizotia abyssinica	Asteraceae	29<br>45.3%	35 54.7%	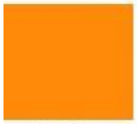	Neon orange Mod
Sesamum indicum	Pedaliaceae	0<br><br>0%	1<br><br>100%	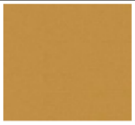	Beautiful Blonde
Vicia faba	Fabaceae	0<br><br>0%	1<br><br>100%	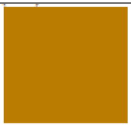	Breezy Blonde
Zantedeschia aethiopica	Araceae	0<br><br>0%	11 100%	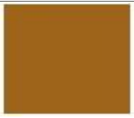	Golden Honey

18 species of ~~pollinators of~~pollinators of G.abyssinica were ~~recored~~recorded and identified which are classified under 12 ~~familes ,83.2~~families, 83.2 % were *Apis mellifera* where as 8.49 % were *Musca* ~~.~~*domestica* (Table 2). Honeybees visited ~~flowers of~~flowers of *G.abyssinica* for nectar and pollen as closely observed visually which is similar to results reported by (Vanitha and Raviprasad 2019). Around 83.2% pollinators were belongs to family Apidae which were honeybees mainly in morning as well as in afternoon than all pollinators this is in line with the ~~rports~~reports of ~~(~~Munyuli (2012). Foraging speed in terms of time spent on each cashew flower varied between bee species(Herman 2015). Pollinators other than honey bees are also extremely valuable although their value is difficult to estimate(Mcgregor and Bean 2009). Time has significant effect on species ~~distributions with~~distributions with p- value of < 0.001. A total of eighteen insect species representing six families visited Niger flowers ~~similary~~similarly ~~thse~~these species of families mentioned in table 2 were visitors of Niger in our ~~experiment(~~experiment (Veereshkumar K. M. Kumaranag 2021).

Formatted: Font: Italic

Commented [Ofijan16]: Rewrite it, not clear

Commented [Ofijan17]: See and rewrite again. Not standard citation

Table 2: Scientific name of ~~pollinators ,their~~pollinators, their relative abundance and ~~total species~~total species occurred ~~at time~~at time classified as below

Formatted: Font: Not Italic

Scientific name of pollinators	Families of pollinators	Relative abundance of each species at a given time below						Total %
		**10:20-11:50 AM**	**1:15-2:45PM**	**2: 45-4:15 PM**	**4:155:45PM**	**7:20-8:50 AM**	**8:5010:20 AM**	
Amata alicia	Erebidae	0.12	0	0	0	0	0	0.12
Anthene princeps	Lycaenidae	0.06	0	0	0	0	0	0.06
Apis mellifera	Apidae	20.7	17.91	12.61	2.47	8.72	20.7	83.20
Calliphora vicina	Calliphoridae	0	0	1.00	0.18	0	0	1.18
Cosmetra tumulata	Tortricidae	0.06	0	0	0	0	0	0.06
Epiphora fournierae	Saturniidae	0	0	0	0	0.06	0	0.06
Eupeodes luniger	Syrphidae	0.35	0.41	0.29	0	0.41	0.41	1.89
Graphomya maculata	Muscidae	0.058	0	0	0	0	0	0.06
Lexias pardalis	Nymphalidae	0.06	0.06	0	0	0	0	0.12
Meliscaeva auricollis	Syrphidae	0.59	0.18	0	0	0	0.47	1.24
Musca.domestica	Muscidae	1.06	1.36	1.00	0.53	2.89	1.65	8.49
Myathropa florea	Syrphidae	0.18	0.29	0.24	0	0.82	0.82	2.36
Phalaena euphemia	Nymphalidae	0	0	0	0	0	0.12	0.12
Platycorynus dejeani	Chrysomelida	0	0	0.06	0	0	0	0.06

Commented [Ofijan18]: Why different font color? Make similar and standard color for all

Syrphus ribesii	eSyrphidae	0	0	0.71	0	0	0	0.71
Vespula vulgaris	Vespidae	0.06	0	0	0	0	0	0.06
Xylocopa longespinosa	Apidae	0	0	0.06	0	0	0	0.06
papilio dardanus	Papilionidae	0.06	0.12	0	0	0	0	0.18

Time has significant effects on these distribution of pollinators species and -their families -at P<0.001. Peak bee activity was recorded between 10:20 -11: 50 AM ;,Similarly, to our findings a study conducted by Vanitha and Raviprasad (2019) showed that peak bees activity was recorded between 11.00 and 13.00 hrs and this has the highest species richness which was 16 (Table 3). Which will increasewill increase primary productivity and this implies there is great pollinatorspollinator's diversity for pollination pursposepurpose which will play a key role in crop yield production and productivity for this area and which is key results for pollinators diversity conservation. Where as the lowest species richness 8 was recorded between 8:50-10:20AM. 1.74 .The highest species diversity was recorded between 2: 453:15 PM where as the highest eveness of 0.68 was recored between 2: 45-3:15 PM.

Commented [Ofijan19]: Please rewrite it again. Make short and precise sentences

Table 3**: Pollinators diversity,richness and eveness at agiven exposure time**

**Time**	**Diversity**	**Richness**	**EvenessEvenness**
1:15-2:45 PM	1.0012726	12	0.4029417
10:20 -11: 50 AM	1.2542643	16	0.4523802
2: 45-3:15 PM	1.7380259	13	0.6776063
4:15-5:45 PM	0.6365142	13	0.2481586
7:20-8:50 AM	0.8484737	14	0.3215064
8:50-10:20 AM	0.61630450.61	8	0.2963798

Commented [Ofijan20]: Write by 2 digits only

Commented [Ofijan21]: Why different color?

The highest species richneesrichness-, diversity and evenessevenness were seen at plots mentioned below in the morning than in the afternoon (Table 4). In the first cycle more species richness were seen at plot A1 ,D1 and E1 and I1 than the others, high diversity were seen at Plots D1,A1 and B1 respectively than the others where as highest evenessevenness were seen at plots B1,D1 and A1 respectively than the others. In the **2nd** cycle species richness were seen at plots D2, A2,B2,F2 respectively , diversity were seen at plots D2,B2,A2 and C2 wehere as species evenessevenness were seen at C2,B2 and A2 respectively. In the 3rd cycle highest species richness were seen at A3,B3,C3,D3,E3,G3 and H3 than the others respectively ,highest species diversity were seen at plots E3,A3 and D3 respectively whereas the highest species eveness were seen at plots E3 than all the others.

In the 4th cycle the highest species richness were seen at plots C4,E4,F4,G4 and I4 respectively than the others , the highest species diversity were seen at plots F4,E4 and I4 respectively where as the highest species ~~eveness~~evenness were seen at plots F4 and E4 than the others. In the 5th cycle the highest species richness were seen at plots C5,D5,and E5 respectively than the others, the highest species diversity were seen at plots C5,G5,H5,E5,D5 and F5 respectively than others where as highest species eveness were seen at plots G5,H5,I5,F5 and C5 respectively than others. In the 6th cycle the highest species ~~richnees~~richness were seen at plots C6,E6 and F6 respectively ,highest diversity were seen at plots E6,C6 and F6 respectively ~~where as~~whereas the ~~eveness~~evenness were seen at plots E6,C6 and F6 respectively than all the others.

Commented [Ofijan22]: Rewrite the whole part. It's confusing part
Not write the whole plots; make short and clear

Table 4: Plots ,pollinators diversity ,species ~~reachness~~richness and ~~eveness~~evenness

1st cycle			
**plots**	**H**	**Richness**	**~~Eveness~~Evenness**
A1	1.21	4	0.88
B1	1.04	3	0.95
C1	0.56	3	0.81
D1	1.24	4	0.89
E1	0.85	4	0.77
F1	0.70	3	0.64
G1	0.76	3	0.69
H1	0.42	3	0.38
I1	0.75	4	0.54
2nd cycle			
A2	0.79	4	0.57
B2	0.81	4	0.59
C2	0.78	3	0.71
D2	0.83	6	0.46
E2	0.20	3	0.18
F2	0.51	4	0.37
G2	0.10	2	0.14
H2	0.39	3	0.35
I2	0.32	3	0.29
3rd cycle			
A3	0.63	5	0.39
B3	0.34	4	0.25

C3	0.48	4	0.35
D3	0.60	4	0.43
E3	0.95	4	0.86
F3	0.43	3	0.39
G3	0.31	4	0.23
H3	0.46	4	0.33
I3	0.29	2	0.41
4th cycle			
A4	0.00	1	NaN
B4	0.13	2	0.19
C4	0.33	4	0.24
D4	0.40	3	0.36
E4	0.72	4	0.52
F4	0.73	4	0.53
G4	0.55	4	0.39
H4	0.50	3	0.46
I4	0.67	4	0.48
5th cycle			
A5	0.00	1	NaN
B5	0.00	1	NaN
C5	0.99	5	0.61
D5	0.54	5	0.34
E5	0.77	5	0.48
F5	0.52	2	0.75
G5	0.91	3	0.83
H5	0.81	3	0.74
I5	0.51	2	0.74
6th cycl e			
A6	0.00	1	NaN
B6	0.00	1	NaN
C6	0.56	2	0.81
D6	0.00	1	NaN
E6	0.66	2	0.95
F6	0.56	2	0.81
G6	0.00	0	0.00
H6	0.00	0	0.00

I6	0.00	0	0.00

Letters from A1 up to I1 represents plots 1m*1m in the morning while Letters from A2 up ~~n~~to~~m~~ 12 represents plots size of 1m*1m taken in the afternoon from where insects were ~~tarpped~~trapped by an interval of 10 minutes both in the morning and afternoon for identification.

Conclussion

Results discovered that honeybees fed sugar syrup collected much more pollen than colonies not fed sugar syrup. The proportion of ~~*Guizotia*~~ *G. abyssinica* pollen collected through sugar syrup feeding of colony was greater (62.2 %), compared to the proportion pollen trapped without colony feeding with sugar syrup (37.8%). This indicates that sugar syrup feeding enhances the collection of ~~*Guizotia*~~ *G. abyssinica* pollen and ~~probability~~ probabily the pollination efficiency of honeybees. Therefore, ~~honeybees~~honeybee's pollination services should be included as one of agronomic input to meet the yield of ~~*Guizotia*~~ *G. abyssinica* through pollination management of honeybees**.** 83.20 % of pollinators of ~~*Guizotia*~~ *G. abyssinica* was honeybees. A future study is necessary to pinpoint Honeybees pollination for different crops ~~like different oil crops and horticultural crops and others~~ to increase ~~yield~~ seed quantity and quality by honeybees and using ~~sigar~~sugar syrup for colony feeding as pollination management is important. This is the first novel work using sugar syrup as pollination management ~~of honeybees~~of honeybees for yield increment rather than using only fertilizer. The highest species ~~richnees ,diversity~~richness, diversity and ~~eveness~~evenness were seen at plots~~-~~ in the morning than in the afternoon. Generally ~~knowning~~knowing the status of our ~~pollinatiors~~pollinator's ~~diversity,richness~~diversity, richness and ~~eveness~~evenness will help to do more on species conservation to use ~~use~~ them for crop yield increment ~~throught~~through pollination since 90 % of crops needs insects pollination to solve the problems of food security. Knowing ~~all species~~all species ~~status and~~status and their pollination role for all crops need pollination was recommended for further research.

Funding:

The authors would like to express their deepest gratitude to the Oromia agricultural research institute as financial support with which to conduct this research.

Conflicts of Interest

The authors declare that they have no conflicts of interest regarding the publication of this paper since it is our original work.

Commented [Ofijan23]: How? You have no yield data

Commented [Ofijan24]: Honeybees, honeybees, Honey bees, honey bees; Which one is an appropriate word? And use one word consistently throughout your manuscript

Commented [Ofijan25]: Rewrite , not smart sentences

Commented [Ofijan26]: From where you record the increased yield owing to the honeybee pollination? You are concluding without a collected and analyzed data

Commented [Ofijan27]: This is somebody's data, how you can conclude it

Commented [Ofijan28]: Please rewrite it again, not clear

Acknowledgments:

First and foremost, I am very much thankful to ~~Ethiopian agricultural~~Ethiopian Institute of Agricultural R~~r~~esearch~~ institute~~. I need to acknowledge my wife Dr. Zemzem Ahmed tendered her inspiration while I was writing the paper and collecting data from long distance and finally I need to acknowledge Oromia A~~a~~gricultural R~~r~~esearch ~~institute~~ Institute for financial support for this work in all aspects.

References

Azzu, Gretchen leBuhn sam Droege ed connor Barbara Gemmill-Herren nadine. 2016. Protocol to Detect and Monitor Pollinator Communities Guidance for Practitioners Protocol to Detect and Monitor Pollinator Communities.

Cardoza, Yasmin J, Gabriel K Harris, and Christina M Grozinger. 2012. “Effects of Soil Quality Enhancement on Pollinator-Plant Interactions.” *Hindawi Publishing Corporation* 2012.

Carlo H.R. HElP, Peter M.J. HERMAN & Karline SOETAERT Netherlands. 1998. “Indices of Diversity and Evenness *.” Netherlands Institute of Ecology Centre for Estuarine and Coastal Ecology 24(2459): 61–87.

Eeraerts, Maxime, Ruben Vanderhaegen, Guy Smagghe, and Ivan Meeus. 2019. “Pollination Efficiency and Foraging Behaviour of Honey Bees and Non- Apis Bees to Sweet Cherry.” Agriculture and Forest Entomology.

Foundation for Arable Research. 2012. “Crop Pollination.” *Crop pollination*.

Gotelli, Nicholas J, and Anne Chao. 2018. Encyclopedia of Biodiversity Measuring and Estimating Species Richness , Species Diversity , and Biotic Similarity from Sampling Data Provided for Non-Commercial Research and Educational Use Only . Not for Reproduction , Distribution or Commercial Use . Elsevier Ltd. http://dx.doi.org/10.1016/B978-0-12-384719-5.00424-X.

Goudarzian, P, and Erfanifard Sy. 2017. "The Efficiency of Indices of Richness , Evenness and Biodiversity in the Investigation of Species Diversity Changes (Case Study : Migratory Water Birds of Parishan International Wetland , Fars Province , Iran)." Goudarzian P, Erfanifard SY 1(2): 41–45.

Herman, Peter M J. 2015. "Indices of Diversity and Evenness." *Indices of diversity and evenness* (October).

Hoover, Shelley E, and P Lynae. 2018. "Apiculture and Social Insects Pollen Collection , Honey Production , and Pollination Services : Managing Honey Bees in an Agricultural Setting." *Journal of Economic Entomology* 111(May): 1509–16.

Kachhela, H R, and J J Pastagia. 2018. "Effect of Abundance of Pollinators on Yield Parameter of Niger , Guizotia Abyssinica (L . f .) Cass . Grown at Different Dates of Sowing." *Journal of Entomology and Zoology Studie* 6(5): 2393–96.

Kumar, G N Veera, E Gangappa, and P Mahadevu. 2006. "Studies on Floral Biology and Autogamy in Niger [Guizotia Abyssinica (L . t .) Cass]." 66(2): 131–33.

Lau, Jenny Y Y et al. 2017. "Time-Dependent Trapping of Pollinators Driven by the Alignment of Floral Phenology with Insect Circadian Rhythms." Time-Dependent Trapping of Pollinators Driven by the Alignment of Floral Phenology with Insect Circadian Rhythms 8(June): 1–11.

Mcgregor, S E, and Scarlet Runner Bean. 2009. "Insect Pollination Of Cultivated Crop Plants." Insect Pollination Of Cultivated Crop Plants.

Munyuli, M B Théodore. 2012. "Diversity of Life-History Traits , Functional Groups and Indicator Species of Bee Communities from Farmlands of Central Uganda." Jordan Journal of Biological Sciences 5(1): 1–14.

Nurlaila, Ai, Ika Karyaningsih, Nina Herlina, and Iing Nasihin. 2021. "Diversity of Insect Pollinator on Farmland near to Mount Ciremai National Diversity of Insect Pollinator on Farmland near to Mount Ciremai National Park." *IOP* Conference Series: Earth and Environmental Science PAPER.

Ormann, C Arsten F D, and A Ndrea H Olzschuh. 2013. "Bee Diversity Effects on Pollination Depend on Functional Complementarity and Niche Shifts." *Ecology* 94(9): 2042–54.

Piano, D E et al. 2021. "Food Supply in Honeybee Colonies Improved Kiwifruit (Actinidia Deliciosa Liang & Ferguson) (Actinidiaceae : Theales) Pollination Services Food Supply in Honeybee

Colonies Improved Kiwifruit (Actinidia Deliciosa Liang & Ferguson) (Actinidiaceae : Th.” *ResearchGate* (September).

Sc, G P Chapman B. 2016. “Pollination and the Yields of Tropical Crops : An Appraisal AND THE YIELDS.” Taylor and Francis 5541(June).

Shaden A. M. Khalifa 1,*, Esraa H. Elshafiey 2, Aya A. Shetaia 2, Aida A. Abd El-Wahed 3 et al. 2021. “Crop Production.” *MDPI*: 1–23.

Sharma, A. Getinet and S.M. 2014. *Niger. Guizotia Abyssinica (L.f.) Cass.*

Somerville, Doug. 2012. “Pollen Trapping and Storage.” *FACTSHEET* (May).

Statement, Non-discrimination. 2017. Attractiveness of Agricultural Crops to Pollinating Bees for the Collection of Nectar and / or Pollen.

Steffan-dewenter, Ingolf, and Catrin Westphal. 2008. “The Interplay of Pollinator Diversity , Pollination Services and Landscape Change.” Journal of Applied Ecology: 737–41.

T. Kro¨ mer1, M. Kessler1, G. Lohaus2 & A. N. Schmidt-Lebuhn. 2008. “Nectar Sugar Composition and Concentration in Relation to Pollination Syndromes in Bromeliaceae Nectar Sugar

Composition and Concentration in Relation to Pollination Syndromes in Bromeliaceae.” *Research GateGate* (August).

Thakur, Meena. 2012. “Bees as Pollinators – Biodiversity and Conservation.” *Int. Res. J. Agric. Sci. Soil Sci. The* 2(January): 1–7.

Tolera Kumsa Gemeda, Jilian Li, Shudong Luo, Huipeng Yang, Tingting Jin, Jiaxing Huang, Jie Wu. 2018. “Pollen Trapping and Sugar Syrup Feeding of Honey Bee (Hymenoptera : Apidae) Enhance Pollen Collection of Less Preferred Flowers.” RESEARCH ARTICLE: 1–14.

Vanitha, K, and T N Raviprasad. 2019. “Diversity, Species Richness and Foraging Behaviour of Pollinators in Cashew.” *Agricultural research* 8(2): 197–206.

Veereshkumar K. M. Kumaranag, Dibyendu Deb4 Vasuki V. Belavadi. 2021. “POLLINATION STUDIES ON BOTH FLORET AND CAPITULUM LEVELS IN.” *Vol. 65 No. 1 2021* 65(1): 85–99.

Tewari GN, Singh K (1983) Role of pollinators in vegetable seed production. Indian Bee J 45:51
