# Supplementary material for: Pollen trapping as Honeybees pollination management and identification of dominant pollinators of Guizotia abyssinica (L.f)
Source: PLoS One. 2024 Oct 10;19(10):e0292565. doi: 10.1371/journal.pone.0292565 (PMC11469591; doi:10.1371/journal.pone.0292565)
Supplement: Attachment — Submitted filename: Responce to reviewers of plos one.docx [file pone.0292565.s002.docx]

**Response to reviewers**

**Comments to the Author**

1. Is the manuscript technically sound, and do the data support the conclusions?

The manuscript must describe a technically sound piece of scientific research with data that supports the conclusions. Experiments must have been conducted rigorously, with appropriate controls, replication, and sample sizes. The conclusions must be drawn appropriately based on the data presented.

Reviewer #1: Yes

Reviewer #2: No

**Response** for Reviewer #2:

Please the conclusion has been made based on results of the finding and for replication and sample size objective matters as you can understand from the title it was to pollinate this *Guizotia abyssinica* by fewer numbers of colonies to enhance pollination

2. Has the statistical analysis been performed appropriately and rigorously?

Reviewer #1: Yes

Reviewer #2: No

Response Reviewer #2: we depend on nature our data and Shannon diversity index was used as mentioned in table of pollinators .Mean weight of pollen collected by fed and none fed colonies mentioned and it is not necessary to go for further analysis since it has answered objective aimed for this research. You can further check the results please.

3. Have the authors made all data underlying the findings in their manuscript fully available?

The [PLOS Data policy](http://www.plosone.org/static/policies.action#sharing) requires authors to make all data underlying the findings described in their manuscript fully available without restriction, with rare exception (please refer to the Data Availability Statement in the manuscript PDF file). The data should be provided as part of the manuscript or its supporting information, or deposited to a public repository. For example, in addition to summary statistics, the data points behind means, medians and variance measures should be available. If there are restrictions on publicly sharing data—e.g. participant privacy or use of data from a third party—those must be specified.

Reviewer #1: Yes

Reviewer #2: Yes

4. Is the manuscript presented in an intelligible fashion and written in Standard English?

PLOS ONE does not copyedit accepted manuscripts, so the language in submitted articles must be clear, correct, and unambiguous. Any typographical or grammatical errors should be corrected at revision, so please note any specific errors here.

Reviewer #1: Yes

Reviewer #2: No

Response to Reviewer #2: I am researcher and published many papers and I have checked plagiarism, grammar and spelling please for further check the paper .Thank you

5. Review Comments to the Author

Please use the space provided to explain your answers to the questions above. You may also include additional comments for the author, including concerns about dual publication, research ethics, or publication ethics. (Please upload your review as an attachment if it exceeds 20,000 characters)

Answer: added in documents thank you

Reviewer #1: The manuscript is technically fitted the journals scope and its conclusion is in line with a data. Please make a uniform how to write a citation in the body and reference throughout the manuscript consistently.

Answer good comments added in text thank you.

Reviewer #2: Reviewer Comments.
1. The scientific name of “Guizotia abyssinica” is not written in right form as French naturalists studied the plant and Verbesina oleifera was the first name given to niger. Polymnia abyssinica L was the first botanical description of the niger (Cassini, 1821). In 1905 following the Vienna Botanical Congress, the name Guizotia was conserved, and in 1930 at the Cambridge Botanical Congress the name Guizotia abyssinica (L. f.) Cass. was proposed as the correct name (Getinet and Sharma, 1996).

Answer: *Guizotia abyssinica* (L.f) please I am Botanist I have learned like this thank you

2. The introduction part is too long and ideas are repeated and don’t keep the flow of ideas.

Ok good comments corrected in paper check there please

3. The sub-topic species richness estimation in the introduction part is extra so, make the idea blend with the introduction and keep the flow of the idea. Done in text good comments

4. Most Scientific names are not italicized.

Ok done in text thank you good comments.

5. Starting from the abstract the manuscript have gap problem, citation problem, ideas are repeated, Spelling error, and some sentence or paragraphs are not clear and all are highlighted in green color . answered in manuscript thank you

6. Figure 1. Mounting and labeling of the insect’s specimen for identification didn’t seem the picture of honeybee and it’s not a clear image.

Answer: It is not picture of honeybees it is picture of identified pollinator’s diversity of *Guizotia abyssinica* (L.f) please

7. This manuscript explains the honeybee as a pollinator but in the result and discussion part the author explains other pollinators and I highlighted it in green.

Answer other pollinators diversity are also listed in table but honeybees are dominant as mentioned there in table of pollinators diversity.

8. Poor sentence construction in result and discussion. Answer corrected good comments thank you

9. Most citations are not correctly cited. E.g Steffan-dewenter and Westphal 2008 cited in the introduction are three authors in the reference section which can be written (Steffan et al., 2008). Good observation corrected perfectly since citation changed according guide line of the journal guide line , thank you

10. The manuscript is not submitted based on PLOS ONE author’s guidelines it should include page no, continuous line number, Vancouver reference style, figure and table shouldn’t be found in the main manuscript file. Ok corrected thank you .
